# Direct electrification of silicon microfluidics for electric field applications

**DOI:** 10.1038/s41378-023-00552-w

**Published:** 2023-06-19

**Authors:** Diego Monserrat Lopez, Philipp Rottmann, Gabriel Puebla-Hellmann, Ute Drechsler, Marcel Mayor, Sven Panke, Martin Fussenegger, Emanuel Lörtscher

**Affiliations:** 1 IBM Research Europe - Zurich, Säumerstrasse 4, CH-8803 Rüschlikon, Switzerland; 2 ETH Zürich, Department of Biosystems Science and Engineering, Mattenstrasse 26, 4058 Basel, Switzerland; 3 University of Basel, Department of Chemistry, St. Johanns-Ring 19, CH-4056 Basel, Switzerland; 4 Institute for Nanotechnology (INT), Karlsruhe Institute of Technology (KIT), P. O. Box 3640, 76021 Karlsruhe, Germany; 5 University of Basel, Faculty of Life Science, Basel, Switzerland

**Keywords:** Engineering, Nanofluidics, Microfluidics

## Abstract

Microfluidic systems are widely used in fundamental research and industrial applications due to their unique behavior, enhanced control, and manipulation opportunities of liquids in constrained geometries. In micrometer-sized channels, electric fields are efficient mechanisms for manipulating liquids, leading to deflection, injection, poration or electrochemical modification of cells and droplets. While PDMS-based microfluidic devices are used due to their inexpensive fabrication, they are limited in terms of electrode integration. Using silicon as the channel material, microfabrication techniques can be used to create nearby electrodes. Despite the advantages that silicon provides, its opacity has prevented its usage in most important microfluidic applications that need optical access. To overcome this barrier, silicon-on-insulator technology in microfluidics is introduced to create optical viewports and channel-interfacing electrodes. More specifically, the microfluidic channel walls are directly electrified via selective, nanoscale etching to introduce insulation segments inside the silicon device layer, thereby achieving the most homogeneous electric field distributions and lowest operation voltages feasible across microfluidic channels. These ideal electrostatic conditions enable a drastic energy reduction, as effectively shown via picoinjection and fluorescence-activated droplet sorting applications at voltages below 6 and 15 V, respectively, facilitating low-voltage electric field applications in next-generation microfluidics.

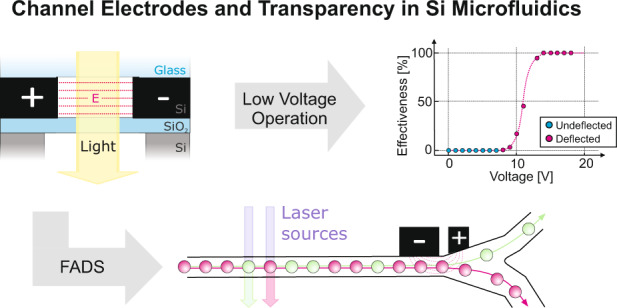

## Introduction

Liquids in constrained geometries possess unique properties due to surface forces^1,2^, such as surface tension, surface drag, or energy dissipation through the nearby surface, which dominates over the volumetric forces. With the advent of microfluidic systems, it has become possible to control and manipulate small quantities of fluids at the nanoliter or subnanoliter level^3^. This technology enabled novel applications with great advances in the fields of chemistry, biochemistry, nanotechnology, and biotechnology^4,5^. For instance, in biotechnology, a broad variety of microfluidic systems was developed that enabled the miniaturized reproduction and integration of critical processes in biological experiments, such as compartmentalization, long-term cell culturing and monitoring, cell separation or single-cell analysis, culminating in complex applications, such as pharmaceutical drug testing on the single or multicell level^6^. Similarly, microfluidic systems are beginning to revolutionize the field of protein engineering, in which a few improved variants of important proteins, such as enzymes, antibodies, or small peptides, are isolated from a pool of less desirable species. Here, compartmentalization on a small scale plays a critical role, as each compartment allows the attainment of one physical linkage between the genetic information for a protein (DNA) and the encoded protein itself, whose properties need to be assessed^7^. Since the identification of one improved protein variant typically requires sorting thousands to millions of variants, fast high-throughput screening (HTS) systems are essential, and miniaturization plays a critical role^8^.

The ultimate compartment in biology is the single cell, and correspondingly, many HTS methods revolve around sorting cells that relate improved protein properties in complex biological assay processes to changed fluorescence properties (fluorescence-activated cell sorting, FACS)^9,10^. Single-cell analysis has undergone much progress in recent years^11^; however, cells as compartments in assay design also have distinct disadvantages, such as limited mass transfer across membranes, limited protein amounts inside a single cell (assay sensitivity), or simply toxicity of an assay to the involved cells. Artificial soft-matter compartments have emerged as a popular approach to overcome these limitations^12,13^. They can be generated and functionalized using dedicated microfluidic components or direct chemical methods. This approach defines the subfield of droplet-based microfluidics. By simply combining two fluid paths into one, water-in-oil emulsion droplets with tailored dimensions down to pico- and subpicoliter volumes can be generated^14^. This well-defined compartmentalization strategy to incorporate various reagents into small, isolated volumes reduces the total amount of material necessary, decreases cost, and enables fully compartmentalized reactions or reactions with a defined exchange of intermediates, catalysts, or products. Connecting any biochemical assays in these droplets to a fluorescent readout enables the use of fluorescence-activated droplet sorting (FADS) systems, providing screening and sorting of more than 1000 droplets per second. FADS has been used successfully to improve enzymes, antibodies, DNA, and DNA-modifying proteins^15,16^.

### Device implementation using PDMS

**Fig. 1:**
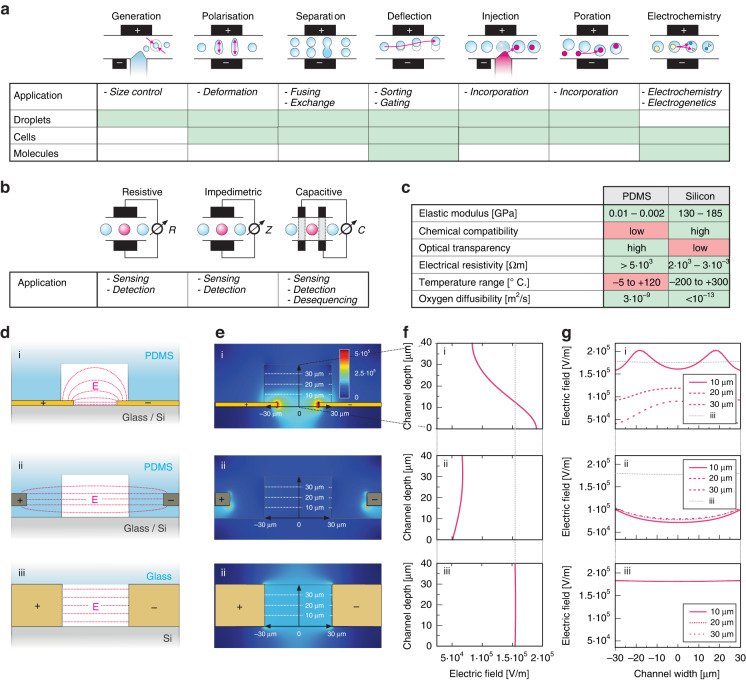
Applications and embodiments of electrodes arranged along a micro- or nanofluidic channel to use electric field effects: **a** Influence on microfluidic objects passing through the microfluidic channel, induced by the underlying (di-) electrophoretic, electroosmotic, or electrochemical (including electrocatalytic, electrogenetic, etc.) mechanisms. **b** Detection modalities of objects present within the probing volume of the microfluidic channel, based on changes in the ionic currents, electrical resistance (including tunneling currents), dielectrics/capacitance, etc. **c** Comparison of the PDMS and Si properties that were used as the main material for composing the microfluidic architecture. **d** Schematic, cross-sectional views of the typical architectures (not to scale) with PDMS, glass, silicon and metal (yellow), meltable metals or ionic liquids (olive), or intrinsic or doped semiconductor materials (beige). **e** Qualitative simulations showing the electric field distribution within the microfluidic channels for flat electrodes (top), channel-near electrodes (middle), or channel-embedded, electrified walls (bottom). **f** and **g** Graphs of the electric field module within the microfluidic channel across different cross-sections for each electrode architecture obtained from the simulations

Most microfluidic chips are fabricated by molding polydimethylsiloxane (PDMS) and bonding the channel containing the PDMS segment to a glass substrate^17^, leading to fully transparent devices. PDMS molding is a simple and inexpensive process and can be repeated several times using the same casting mold. Furthermore, PDMS provides unique properties that are often beneficial for specific applications (see Fig. 1c). Its low mechanical compliance, however, can limit operational pressures, such as those required for operating high-viscosity media, and lead to a pulsing of channel cross-sections. These unwanted effects affect throughput and precision. Therefore, extensive efforts have been made to increase the rigidity of PDMS^18^. Furthermore, PDMS’s gas diffusivity enables trapped air to be removed, but it can potentially affect the results due to CO_2_ diffusion^19^ or vapor loss^20^. Limited lifetime is another limitation of PDMS-based systems since the polymer degrades upon the operation and therefore requires frequent replacement^21,22^. Optical access for readout in transmission geometry for FADS applications can be easily established without further effort through the transparent PDMS and glass upper and lower channel walls. However, the higher autofluorescence of PDMS compared to glass^23^ generates an unwanted background in the acquired optical spectra. Furthermore, as an insulator, PDMS displays limitations when used in applications where electrical fields and conductive sections for the creation of electrodes are needed^24–26^.

### Electric fields as manipulation mechanisms

Different mechanisms have been used to control and manipulate individual cells and droplets^27^, ranging from pneumatic valves^28^ over acoustic waves^29^ and laser-induced heating^30^ to electric fields. Electric fields are mostly used because they are highly efficient at small dimensions and enable a large variety of other tasks to be achieved on the same device, including electrical sensing^31^, droplet fusion^32^, picoinjection^33^, or sorting^24^. Figure 1a schematically shows the various applications that electric fields render possible, together with the objects that can be manipulated by those means. In Fig. 1b, the basic electrical sensing modalities are illustrated but not fully comprehensive. The electrical deflection approach is scalable in terms of the number of such elements and miniaturizable to concentrate electric fields into small volumes. The approach working on the electrical domain is directly compatible with the control electronics that can, in principle, even be integrated into the chip.

To achieve microfluidic applications involving electric fields (to enable some of the applications depicted in 1a), at least two electrodes need to be arranged across or along the microfluidic channel such that electric fields are present within the channel. While an optimal placement of electrodes is apparent, as illustrated in 1a, the physical attainment is not straightforward and, therefore, subject to extensive research efforts. Thin-film electrodes are widely used, patterned on a glass^24^ or a silicon chip (Fig. 1d i), and subsequently bonded to a PDMS chip containing the channels. This approach provides submicron feature sizes with a lateral, in-plane separation only limited by the lithographic technology used. Since the electrodes do not protrude into the channel itself, the electric field orthogonal to the thin film plane rapidly decays, causing large field gradients and inhomogeneities over the entire channel cross-section with local hotspots, as illustrated in the upper rows of Fig. 1e–g. More symmetrical geometries can be obtained by using auxiliary channels in the PDMS corpus that are filled with a conductive material (Fig. 1d ii), including low-melting point solders^34,35^, silver paste^25^ or ionic liquids such as salt water^26^. Auxiliary channels are similarly facile to fabricate (e.g., by drilling holes or using double-sandwich PDMS structures) and provide electrodes at a height similar to or even higher than the channel itself^36,37^. Due to fabrication tolerances, the electrodes cannot be placed arbitrarily close to the microfluidic channel. This approach is illustrated in the middle rows of Fig. 1e–g. An alternative to the two methods described above is the addition of metal-PDMS composites prior to the microfluidic channels^38–40^; however, this method has the drawbacks that the conductivity is difficult to control and the close placement of the electrodes to the channel needs accurate alignment. A variety of other methods exist, ranging from glass-silicon-glass sandwiches^41^ to ion implantation via a shadow mask^42^, all with severe limitations that prevent widespread usage. New low-cost approaches, such as using conductive ink in thermoplastic-based microfluidic devices, are being explored, and despite their advantageous rapid fabrication, these electrodes cannot be placed close enough to the fluidic channel; thus, an operation is still in the kV range^43^.

None of the above methods used to integrate the electrodes into microfluidic systems achieve homogeneous electrostatic field distributions and sufficient long-term stability. Consequently, the devices require high voltages, often in the hundreds of Volts regime, to operate, simultaneously causing degradation under the high electrical load due to the diffusion of materials and drifting geometries. Since these aspects can be accepted for some applications, there is a large number of techniques that drastically benefit from improved performance to increase precision, yield, throughput, and efficiency while lowering energy consumption and degradation to enable long-term operation. In this regard, silicon as a basic microfluidic channel material instead of PDMS, sealed by a bonded glass wafer, was an attractive concept, especially since it was already used for integrated DNA analysis several decades ago, indicating the general biocompatibility of silicon and biological macromolecules^44^. Due to the unique properties that silicon provides in terms of mechanical compliance and chemical inertness, which can potentially enable PDMS-based microfluidics to be outperformed (see Fig. 1c), here, we present a novel fabrication approach that solves the inherent optical access issue of the underlying silicon platform and further enables 3D electrodes to be placed in closest proximity to the microfluidic channels^45^. Our self-aligned process developed directly places the electrodes at the channel walls without the need for any additional alignment and in close lateral proximity to each other. As a result, we demonstrate the full operation of a FADS device that is characterized not only by a significantly reduced operating voltage due to improved electrostatic field distributions but also benefits from silicon’s characteristics regarding reproducibility and long-term stability. Furthermore, we demonstrate the advantages of nearby electrode placement by developing microfluidic devices for droplet picoinjection at low voltage, as described in the following.

## Results and discussion

### Silicon-on-insulator microfluidic platform

**Fig. 2:**
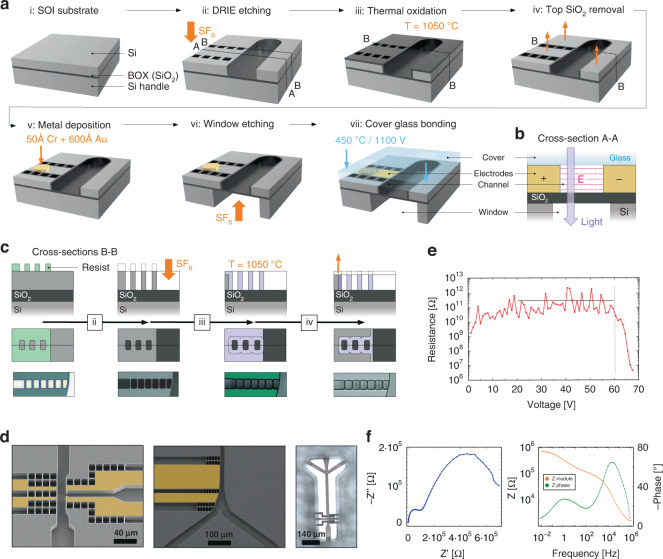
Fabrication route and electrical characterization: **a** Schematic 3D representation of the simplified fabrication steps to create electrically insulated wall segments along a microfluidic channel and an optical transmission window across the channels. Each graphic corresponds to the resulting structure after the labeling process is performed; the intermediate photolithography steps are not represented but described in detail in the Materials and Methods section. **b** Cross-sectional view (A-A) of the resulting device stack. **c** Detailed fabrication schematics across the B-B cross-section of the fabrication of fully insulating segmentation patterns (upper and middle rows), illustrated with optical white-light microscopy images of the real devices fabricated (bottom row). The photoresist used for pattern transfer through DRIE is shown. **d** SEM images of functional structures targeting picoinjection and sorting right after pattern etching (left and middle) with an overlay of electrically addressed segments (beige) and optical microscopy image (right) acquired through the transmission window at a picoinjection structure (viewed from the bottom through the entire handle wafer). **e** Resistance between two adjacent electrodes under a DC load, showing an average value of approximately 340 GΩ (for voltages between 20 and 60 V) and a breakdown at ~60 V. **f** Nyquist plot (left) and Bode plots (right) for electrochemical impedance spectroscopy measurements for the same electrodes as before, using a KCl solution as the electrolyte

To overcome the abovementioned barriers for using silicon in optical and electrical microfluidic devices, to the best of our knowledge, for the first time, we introduce silicon-on-insulator (SOI) technology for microfluidics. SOI substrates were originally developed for the semiconductor industry to improve the electrical performance of electronic devices, namely, silicon-based transistors. This concept relies on reducing the parasitic capacitance of the thin oxide layer by replacing it with a much thicker and, therefore, electrically better insulating oxide layer in the SOI stack. SOI substrates are available in different dimensions and always consist of a (thick) Si handle wafer, a buried oxide (BOX) layer (of variable thickness), and a (thin) Si device layer on top with arbitrary electrical conductance, as shown in Fig. 2a i. The production of these substrates is either based on a separation step induced by the implantation of oxygen, direct wafer bonding or a combination of bonding and exfoliation^46^. For our purpose, the bonding approach provides variable device layer thicknesses that directly match conventional channel depths of a few to some tens of microns, as typically used in microfluidics. Much thinner device layers are currently being used in electronic manufacturing, and the novel approach presented here could also be used for these thin nanofluidic devices. The pristine SOI material stack is used to create self-aligned channels, well-defined electrodes, and transmission viewports for optical experiments.

While etching channels into the device layer is straightforward using deep reactive ion etching (DRIE) or any other etching process^47^, creating electrode pairs in the silicon device layer needs the development of a novel process and its optimization. After channel etching, the BOX, consisting of SiO_2_, not only defined the bottom section of the microfluidic channel but also electrically insulated the two opposing channel walls (assuming that the device is closed by a nonconductive glass cover). Hence, the main challenge remaining was to create an electrical insulation segment in the device layer and along the channel walls without compromising the integrity of the channels. We solved this by simultaneously etching, upon the creation of the channels themselves, additional perforation patterns that pointed orthogonally away from the channel direction, as shown in Fig. 2a ii. Since the channel and perforation pattern were lithographically defined by the same mask, they were considered to be “self-aligned.” This concept eliminated any additional alignment step and enabled arbitrary and nearby placement of the electrodes along the channels, which was crucial, for example, for picoinjection, as discussed later. Since there was a gap between the channel and the first perforation element, this last silicon segment acted as a bullhead to seal the channel toward the perforation pattern. Now, to cause the silicon segments remaining in between the perforation elements to be electrically insulating, an oxidation process was used; upon thermal oxidation, all open Si surfaces of the device layer exposed were converted into SiO_2_ (see Fig. 2a iii), including the entire top surface. Thermal oxidation of silicon is a well-known process in the semiconductor industry, whereby oxygen from the environment is incorporated into the silicon corpus via its exposed surfaces at very high temperatures (*>*1000 °C). By the incorporation of oxygen, silicon expanded in volume upon undergoing a chemical transformation to SiO_2_. Since the classical Deal and Grove’s Model^48^ explained in detail the kinematics of silicon oxidation for various conditions, ~0.44 Å of Si was used to obtain 1.0 Å SiO_2_^.^ Notably, thermal oxidation was a self-limiting process since the growing SiO_2_ layer reduced the oxidation speed itself due to its low oxygen diffusivity, leading to longer process times for achieving thick oxide layers^49^. Hence, the spacing between the perforation patterns needed to be selected accordingly. Upon optimization and by slight overoxidization, the pitch of the perforation elements could be selected in such a way that all silicon in between the perforation elements was fully converted into SiO_2_. Figure 2c schematically shows this process in a cross-sectional and top view along the perforation segments (upper two rows) and illustrates it directly via optical microscopy in the lower row from the photolithography step to create the perforation pattern and the later DRIE step to etch the silicon to its thermal oxidation. After that process, the entire perforation line became electrically insulated based on the repeating sequence of air gaps and oxide bulkhead elements present. For the present case, we used rectangular elements of 10 × 15 µm^2^, with a separation of 2 µm, starting at a distance of 2 µm from the channel. To fully oxidize the entire Si segments in between the perforation openings and to account for the more constrained oxygen access therein, we used an etching time that corresponded to ~1.2 µm oxide formation on a free surface. Due to this nominal overetching, the insulating segments overlapped, as shown in Fig. 2c iii. Furthermore, the thermal oxide intentionally covered the channel walls, thereby insulating the electrode body from the fluid medium to prevent unwanted electrochemical reactions from occurring.

Last, the oxide layer created on top of the device layer through thermal oxidation needed to be completely removed (Fig. 2a iv) to enable anodic bonding to a glass cover to close the microfluidic channel. Complete removal was key for proper sealing of the channel. Notably, the oxide on top of the device layer was irrelevant to the electrical isolation of the electrodes or channel. However, robust anodic bonding with the borofloat glass was only ensured by exposing a clean and residual-free silicon surface. Due to the considerable thickness of the oxide layer (~1.5 µm), most was removed by a reactive ion etching (RIE) process, while the last hundreds of nanometers were etched away using a buffered hydrofluoric acid (BFH) dip to ensure a smooth final surface for stable bonding. Since this last step simultaneously removed the device layer’s native oxide, metal pads were subsequently deposited via gold evaporation, with a previous photolithography step to pattern the pad size and position to create low-ohmic contacts to the electrode layer (Fig. 2a v). Our entire fabrication approach was submitted for patent protection^45^.

Furthermore, the layered structure of SOI substrates with the intermediate and fully transparent BOX layer was ideally suited to support optical access. At various points of interest in the device layout, e.g., where viewports were needed to monitor droplet generation, picoinjection, sorting, etc., the handle wafer could be locally removed to enable optical transmission. This removal was performed via DRIE (Fig. 2a vi), a process that selectively stops on the SiO_2_ surface. Since the BOX defined the bottom channel wall, it needed to be sufficiently thick to offset the pressure applied to the microfluidic channel. For the present case, we selected a BOX thickness of 2.0 µm, which was sufficiently stable for practical operation. Finally, the device was completely sealed by anodic bonding of a top glass wafer (Fig. 2a vii step). The resulting device was fully transparent across some channel sections, enabling visualization in full transmission geometry with a final device cross-section, as shown in Fig. 2b. Figure 2d shows examples of the picoinjection structures and sorting elements, where the image on the right depicts an optical microscopy image acquired from the bottom of the device through one of these opened viewports.

Focusing again on the electrodes created directly at the channel’s walls (Fig. 1d, bottom panel), the resulting electrical fields are very homogeneous across the entire channel cross-section, as simulated in the bottom panel of Fig. 1e. Since the electrodes are very near to the channel but do not protrude into it, the resulting electric field strengths are always much higher than those created by auxiliary channels acting as electrodes. Furthermore, they do not show unwanted hotspots or strong field gradients, as is the case for thin-film electrodes. Figure 1f, g qualitatively compares the electric fields present for the various electrode implementations discussed.

While these properties appear very promising in theory and in simulation, the quality of the electrical insulation created by the local oxidation and the resulting SiO_2_/air-gap bridges between adjacent electrode segments needed to be properly assessed by conducting an electrical characterization of real devices. Figure 2e shows excellent resistances of 10^10^ to 10^12^ Ω for DC voltages up to 60 V, with an average value of ~340 GΩ. For DC loads exceeding 60 V, the resistance dropped, most likely due to leakage paths with the top Borofloat 33 glass cover, an effect that could be prevented by coating the cover with an additional oxide layer, if needed. Since most devices were operated in AC mode, electrochemical impedance spectroscopy was performed using a KCl solution as the electrolyte. Figure 2f shows the measured impedance (both the Bode plots and the Nyquist plot); these results matched the expected behavior for a pair of well-insulated electrodes^50^. These results not only confirmed very good insulation between the silicon-embodied electrodes but also exhibited behavior similar to metal electrodes expected under these operational conditions. For the case where the devices were fabricated to provide an electric field and current flow, the SOI wafers could be doped to increase the conductance almost to the full-metallic range, if needed. Locally, the oxide toward the channel could then be removed entirely, and the electrode surface terminated with another conductive layer in an additional processing step, such that charge carriers could be supplied to the media inside the channel using the very same self-aligned fabrication approach, could be used for electrochemical experiments, protonation, etc.

### Mounting and interfacing SOI microfluidic devices

**Fig. 3:**
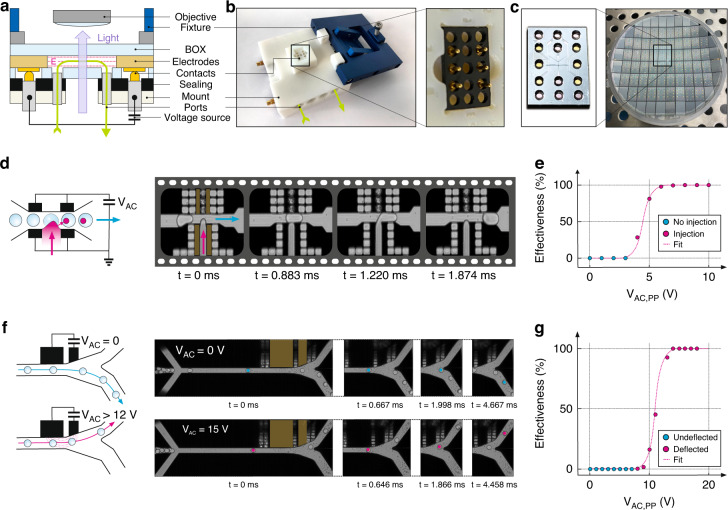
Experimental setup and demonstration of low-voltage droplet injection and deflection: **a** Schematic view of the experimental setup with fluidic ports, electrical contacts, and optical access through the device. **b** Fluid and electrical interfacial mount (left) with sealing and electrical contacts (right). **c** Single device (size 12 mm × 8 mm) with six electrical pads and 4 + 4 fluidic ports (left), fabricated on a full 4”-wafer level containing 56 devices. **d** Droplet injection with two electrode pairs (left) and a time series of images acquired with a high-speed camera along the channel upon injecting small portions of liquids into droplets (right). **e** Voltage-dependent effectiveness of liquid injection into droplets. **f** Droplet route deflection by dielectrophoretic forces created via an electric AC field present at the junction (left), and high-speed camera acquired time series of images with droplets passing the fluid-dynamically more favorable lower route of the asymmetric junction (undeflected) at V_AC_ = 0 V (right, upper panel) and deflected at V_AC*,P P*_ = 15 V @ 30 kHz (right, lower panel). **g** Voltage-dependent effectiveness of the droplet deflection

A large variety of methods exist to interface microfluidic chips with tubing to external liquid-handling hardware (such as pumps, valves, collection vials, analytics, etc.) and to connect to electrical instruments (such as data acquisition cards, voltage, or current sources), ranging from needles to glue-on ports and gasket-based mounts^51^. To take advantage of the rigid SOI–glass platform with well-defined form factors in the submicron regime, we mechanically clamp the chip and a gasket against a 3D-printed mount with the lid, as shown in Fig. 3b, considering the orientations of the electrical and fluidic connections (Fig. 3a). If the operation with nonaqueous solutions is needed, the 3D-printed mount can be manufactured in corrosion-resistant metal prints, e.g., titanium or stainless steel. Alternatively, the mount can also be CNC-machined (e.g., in aluminum) and coated with desired materials. The mount design enables expensive SOI chiplets to be kept comparably small (e.g., 8 mm × 12 mm here), resulting in more chips per wafer and thus decreasing the cost per chip. Figure 3c shows 56 chips fabricated on a single four-inch wafer. Due to the design, the interface can sustain high pressures and is compatible with HPLC threads and fittings (commonly used in microfluidic labs), reducing errors and requiring minimal setup time. Electric connections are made by standard SMC connectors, and reliable contact with the chip’s metallic pads is ensured by spring-loaded pogo pins. The bottom mount and the top lid are further equipped with an aperture for improved light coupling to enable optical access in the center region of the chip. This design is fully compatible with inverted microscopy and high-numerical aperture (potentially even oil immersion) objectives due to its low-profile design. The overall concept provides great flexibility to test different chip designs and multiple applications while maintaining an identical port distribution layout. Nonetheless, it is scalable in terms of the number of fluidic ports (here 8) and electric connections (here 6) due to variable mechanical forces and the massive lid to hold and press the stack together, enabling more complex and interconnected microfluidic operations on a single chip, as described later.

### Droplet experiments

To demonstrate the advantages and the performance increase expected for our novel electrode implementation concept, two widely used and well-understood processes in microfluidic high-throughput screening (HTS) were selected for benchmarking since they were anticipated to greatly benefit from the improved electrostatics. Picoinjection, on the one hand, requires closely positioned electrodes with opposite polarities to create field gradients for the injection of liquids into droplets (illustrated in Fig. 3d). Droplet sorting, on the other hand, only works when high fields can be applied to the channel to induce sufficiently high dielectrophoretic forces to deflect droplets against the fluid-dynamically preferable flow direction in a Y-type junction (illustrated by two flow paths in Fig. 3f). The two designs were achieved following the fabrication route previously described (with full details in the Materials and Methods section). For direct monitoring of the proper operation, the droplets were imaged via a high-speed camera above an inverted microscope and through the optical viewports in the SOI chip.

#### Picoinjection

An injection is an important microfluidic functionality for various applications in biology and chemistry since it enables the addition of a well-defined quantity of a reagent into a droplet compartment. This task for volumes comparable to or greater than the target droplet is accomplished by droplet merging via electrocoalescence^32,52^, and picoinjection^33^ is typically used for smaller volumes. Here, a microfluidic channel perpendicular to the droplet flow direction is filled and pressurized with a reagent such that a stable meniscus is created, as shown in Fig. 3d. An electric field is then used to trigger the rupture (or on the microscopic level, the depolarization) at the water–oil interface^52,53^, finally leading to an injection of the reagent into the droplet. As the droplet moves away from the electric field region through the hydrodynamic flow inside the channel that is controlled by external means (e.g., pumps), the water–oil interface can stabilize again, enabling the droplet to regain its integrity with the new volume incorporated. The amount of material injected can be adjusted via the pressure levels and the flow rates. Subpicoliter accuracy levels, even at kHz injection rates, have previously been demonstrated, but typically high voltages of 200 V or more are needed, with some exceptions where interface rupture is achieved at lower voltages^52^.

To demonstrate the improved electrostatics of our silicon architecture, we utilized a picoinjection design with four electrodes, one on each side of the reagent supply channel and two on the opposite side, as shown in Fig. 3d. Since the optimal electric field direction was expected to be parallel to the interface of rupture^54^, only the electrodes opposite to the injection channel were biased (with a 30 kHz AC signal), while the other electrodes were grounded. Water-in-oil droplets 20 µm in diameter were generated previously, and an aqueous solution containing ink and a fluorescent dye was used as a reagent for optical analysis during and after picoinjection. Droplets were inserted into the device at a 20 µL/h flow rate, while oil was provided at a 75 µL/h flow rate to cause a separation between the droplets, leading to a droplet rate above 400 droplets/second (≈0.4 kHz). The right panel of Fig. 3d shows a sequence of images acquired by the high-speed camera, demonstrating the injection of a small amount of reagent into a larger droplet. Statistics on different voltages were acquired, and videos containing at least 300 droplets were recorded. These videos were manually analyzed to count the number of successful injections to calculate the percentage of successful events (referred to as “picoinjection effectiveness”), represented by the circles in Fig. 3e. As shown by the sigmoidal fit to the experimental data (solid line), the effectiveness of the picoinjection reached 100% for *V*_*pp*_ > 6 V. The injection threshold at which injection began to occur was found to be as low as 4 *V*_*pp*_. Videos for the experiments at different voltages can be found in the supplementary information. This demonstrated the drastic improvement the nearby electrode placement caused on the injection mechanism compared to the literature, where similar channel geometries and operating conditions required supply voltages several orders of magnitude higher^55–57^.

#### Deflection

Separating cells or droplets with desired characteristics from other ones is a basic task with very high relevance in microfluidics. As described in the introduction, FACS and FADS devices achieve high sorting speeds and efficiencies by deflecting (or not deflecting) specific cells or droplets into a sorting channel while other droplets are transported toward the waste channel. Actuation of droplets is possible via dielectrophoresis^58^, for which the high dielectric contrast, e.g., between oil (*c* ≈ 10) and water (*c* ≈ 80) in water-in-oil droplets, creates a force proportional to the strength and gradient of the electric field applied. Since the first demonstration by ref. ^24^, dielectrophoresis has been widely used for droplet sorting applications. In contrast to picoinjection, however, typical voltages are comparably high, ranging from 600 *V*_*pp*_^59^ to 1 *kV*_*pp*_^25^. These settings need a voltage amplifier or high-tension electrical equipment, which causes the setup to be expensive and complex in terms of safe operation and shielding but limited in the operational speed achievable for switching high-amplitude voltages.

For our proof-of-concept devices, an asymmetric design was selected where droplets were by default directed to a wider waste channel in the absence of electrical fields, with the narrower sorting channel placed on the side (Fig. 3f). The pair of electrodes was located on the deflection side, as shown in the middle panel of Fig. 2d. To test the deflection performance of the design, droplets were inserted similarly to the picoinjection experiment previously described. Different conditions were tested by varying *V*_*pp*_, leading to the acquisition of videos containing 200–400 droplet sorting events. As described, we analyzed the percentage of droplets that were directed to the sorting channel in the presence of an electric field as desired, referred to as “deflection effectiveness.” Figure 3f shows the images of the two binary scenarios, captured with the high-speed camera; the upper row shows the case where both electrodes were maintained at 0 V such that all droplets were directed to the waste channel. In the lower row, once a sufficiently high bias was applied to the electrodes, all droplets were deflected and left the junction toward the sorting channel. Figure 3g shows the measurement data as circles and a sigmoidal fit to the experimental data as a solid line. The voltage sweep indicated that at *V*_*pp*_ > 14 V, all droplets were deflected, with an onset taking place below 10 V. Videos for the experiments at different voltages can also be found in the supplementary information. As in the case of picoinjection, these operation conditions represented a drastic decrease of two orders of magnitude in supply voltages over state-of-the-art concepts achieved in similar devices^25,59^. Our result was achieved by placing the electrodes directly at the channel walls and reducing the lateral spacing between them to further increase the field gradient. These voltages could be achieved under standard laboratory conditions without high-voltage circuitry and related safety measures. In principle, these voltages could be supplied via the USB port of a standard PC or high-speed data acquisition cards, causing these sorting devices to be all-purpose and deployable.

#### FADS

**Fig. 4:**
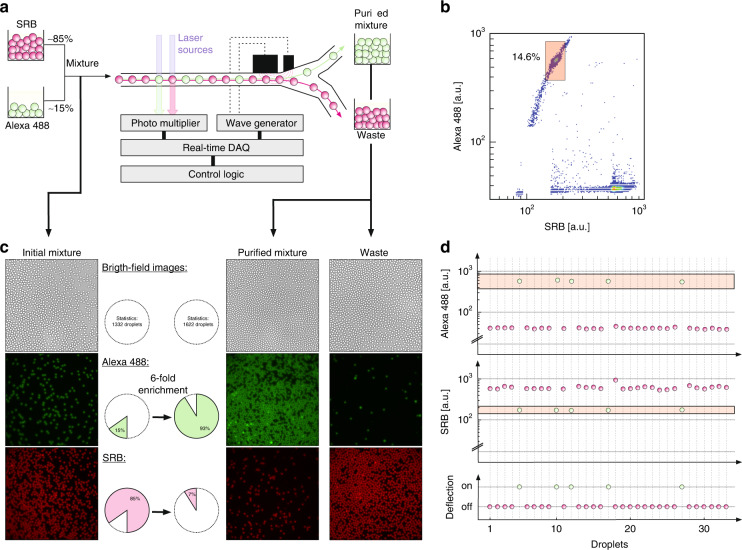
Experimental setup and results for fluorescence-based droplet sorting: **a** Schematic view of the experimental setup with laser sources to induce the fluorescent signal acquired via a photomultiplier to control the voltage pattern applied to the deflecting electrodes via real-time DAQ (FPGA) and control logic. A solution containing ~15% droplets containing Alexa 488 dyes and ~85% droplets containing SRB dyes is analyzed. **b** 2D scatter plot of the droplet population with the red fluorescence signal of the individual droplets on the X-axis (SRB) and the green fluorescence signal of the droplets on the Y-axis (Alexa 488). The red rectangle represents the sorting set to sort out the green fluorescent population. **c** Bright field image comparison of the droplet population before (left) and after sorting (right), including the purified mixture collected from the sorting top channel and the waste collected from the waste bottom channel. **d** Time series of fluorescence intensity measured for certain sequences of droplets and the corresponding sorting activation

Due to this drastic voltage reduction achieved by channel wall-embedded and closed electrode pairs, we further aimed to demonstrate full FADS operation. To create FADS microfluidic devices, the following main components needed to be implemented in a microfluidic platform: (i) a feed channel with optical access to the objects to be screened (cells or droplets), (ii) a delay line, (iii) a deflection mechanism, and iv) collection and waste channels. A general scheme of a FADS device is shown in Fig. 4a. The delay line is a simple extension of the feed channel, required to account for the timing sequence among the acquisition of the optical read-out signal (mostly achieved by photomultipliers), signal evaluation and processing time, as well as the control and delay of the cell or droplet deflection system, e.g., via wave generator. This timing can also be slightly tuned by adjusting the flow rate, which needs to be as high as possible for HTSs. Signal evaluation and processing are usually performed by real-time data acquisition (DAQ) systems with built-in processing capabilities and output channels. Our system uses two different laser sources (488 and 561 nm) for the excitation of the fluorescent species. The laser beam is focused in the middle of the microfluidic channel, and upon excitation of the fluorescent molecules within the droplet, a fluorescent emission signal is produced. The fluorescence signal is redirected through the objective to a photomultiplier tube. A Red Pitaya DAQ is connected to the PC and controlled by the DropSort program. With DropSort, thresholds and sorting criteria can be manually set and sent to the DAQ, which performs adjustments in real-time. Therefore, whenever a droplet fulfils the desired fluorescence characteristics based on the sorting criteria, the DAQ triggers the function generator to create a sorting AC pulse that is applied to the electrodes in the microfluidic device. If a droplet does not meet the required fluorescence characteristics, the DAQ does not trigger any signal, and the droplet remains on its trajectory toward the waste channel. Aside from the acquisition of the fluorescence signals, we monitor the sorting process via a high-speed camera through another optical window.

As a rational test bed, a droplet population consisting of 15% green (Alexa 488) and 85% red (SRB) fluorescent droplets was created and inserted into the sorting microfluidic system at a rate of over 600 droplets/second (0.6 kHz). The DropSort program detected and plotted the two droplet populations in a 2D scatter plot, as shown in Fig. 4b. As expected, two distinct droplet populations could be detected, enabling droplets with increased green fluorescent signal (red rectangle) to be selected amounting to 14% of the total population. The electrical deflection amplitude was set to 30 *V*_*pp*_ at 30 kHz and activated by 0.8 ms long, rectangular trigger pulses from the DAQ. After sorting the green fluorescent droplets based on the thresholds defined by the transparently filled red box in Fig. 4b, images were acquired of the sorted and unsorted populations and quantitatively analyzed with image recognition software, as shown in Fig. 4c. As expected, the initial starting population contained primarily red fluorescent droplets (85%), whereas the sorted population contained primarily green fluorescent droplets (93%, see Fig. 4c). Thus, successful sorting of the green fluorescent droplet population with minimal supply voltages enabled a sixfold enrichment after one round of sorting. Videos of this sorting experiment can be found in the supplementary information, where showing how only certain droplets (green ones) were selectively directed toward the sorting channel (although fluorescence could not be recorded by the camera). The remaining 7% red fluorescent droplets still detected in the sorted population could not be explained by only the statistical intensity distribution outside the desired window or spectral cross-talk in the photo multiplies. Instead, the deflection into the collection path occurred in the case of two adjacent droplets, where the triggered deflection of the desired droplet erroneously attracted another droplet at the same time since it was not properly separated. When neglecting this effect, which was inherent to high-speed FADS, and by comparing raw data channels (the Alexa 488 and SRB fluorescent signals as well as the deflection voltage) and high-speed images acquired at the respective time sequences, the two-droplet cases could be sorted out on the single-droplet level. Our data treatment then provided FADS sorting efficiencies close to 100%, as shown for selected traces in Fig. 4d. Notably, the excellent sorting results at high speeds and drastically improved voltages in a not fully optimized geometry impressively demonstrated the high potential of our new SOI platform. However, FADS operation has been reported at rates of ~1 kHz^60,61^ (high throughput) or even 2 kHz (ultrahigh throughput)^62^, which were above the 0.6 kHz tested for our SOI-based system. Thus, we anticipated that an improved microfluidic design running under optimal operating conditions could significantly even exceed these very good voltage results and provide operation at higher droplet rate values.

## Conclusion

For our study, SOI technology was introduced for the first time in microfluidic applications, enabling arbitrary electrode segments to be incorporated directly into the device layer through a novel fabrication approach with optical viewports for transmission experiments. The direct electrification of channel walls led to ideal electrostatic conditions with highly homogeneous fields present across the channel. Considerably lower supply voltages were demonstrated for electric field applications, including cell and droplet manipulation. Notably, injection and deflection voltages below 6 *V*_*pp*_ and 14 *V*_*pp*_, respectively, were achieved in real devices, providing a reduction of almost two orders of magnitude over current state-of-the-art devices. These low voltages were accessible by standard signal generators or even PC components, causing the devices to be more easily handled and supporting their widespread usage. For high-throughput screening, sorting rates were not typically limited by optical detection or signal processing but rather by the rise time of the electrical deflection voltages causing false-positives^38^. Therefore, the reduced voltage amplitudes in our concept removed the bandwidth limitations of both high-voltage amplifiers and enabled enhanced operation. The SOI-based devices were further shown to have excellent mechanical rigidity and compliance as well as high chemical compatibility toward corrosive solvents, leading to unprecedented long-term stability and novel applications. Due to the low supply voltages and long-term stability without any sign of fatigue even under high load and high-pressure operation, our new approach could have a large impact on high-throughput screening microfluidic applications, enabling increased throughput and improving yield and longer operation. Sorting a higher number of protein variants could lead to a breakthrough, e.g., in protein engineering, since higher thresholds could be reached where advanced applications become feasible. In addition to basic research, the devices enabled fundamentally novel applications in cell biology, next-generation sequencing, etc., and could even support the discovery of improved enzymes, new drugs, etc.

Silicon manufacturing capabilities yield identical devices that can be fabricated with reproducible characteristics, even at mass-production levels, enabling them to be operated without time-consuming tuning of operational parameters caused by device variations. Furthermore, the technology enables a scalable, high-density integration of other functionalities on a single device that is not feasible using other technologies. Hence, the current example potentially represents a starting point of a transition from PDMS- to silicon-based, multipurpose microfluidics since silicon’s opaqueness is no longer a barrier for important applications requiring optical read-out. Importantly, even for our proof-of-concept devices, supply voltages are already compatible with CMOS operation levels. Therefore, seamless and monolithic integration of other electronic, optical, or optoelectrical components (as already fabricated on silicon platforms), such as light sources and detectors (e.g., an III–V semiconductor cointegration on silicon), can potentially lead to miniaturized microfluidic devices in the long term. These can respond in a smart manner and be operated in a lab-on-a-chip approach even outside laboratories for Internet-of-Things applications. This aspect can enable remote diagnostics and smart medical devices with analytical capabilities.

## Materials and methods

### FEA simulations

COMSOL Multiphysics was used to perform finite element analysis of the electric field distribution in microfluidic channels depending on the voltage applied to the electrodes. A 2D model was used to create the different geometries of the cross-sections of interest, and the electrostatics module was used to define the boundary conditions and study conditions.

### Sample fabrication

Silicon-on-insulator wafers 100 mm in diameter, P-doped and with a resistivity of 1–50 Ohm cm^*−*1^, were obtained from University Wafer, with a device layer height of 27.5 µm, a buried oxide (BOX) thickness of 2.2 µm and a handle thickness of 482.5 µm. Borofloat glass 100 mm wafers were obtained from PlanOptik AG, which had patterned holes through the glass by sandblasting. Three chrome masks with designs for the device layer structures, metal pad deposition, and backside etching were fabricated with a Heidelberg DWL2000 laser writer. The GDS file with the exact designs for those three masks can be found in the supplementary material. All design geometric parameter values (channel widths, electrode positions and dimensions, and optical window sizes) for both the injection and sorting devices can be found there. The top of the device layer of the SOI wafer was patterned via photolithography (Microchemicals AZ6612 resist and a Süss Mask Aligner M6) and deep reactive ion etching (DRIE) with an Alcatel AMS 200SE I-Speeder. Subsequently, 1.2 µm of silicon was converted to silicon oxide via thermal oxidation in a furnace at 1050 °C and atmospheric pressure for 28.5 h, such that the entire sections in between the perforation openings become insulating. Then, a thick layer of photoresist (Microchemicals AZ4562) was spin-coated on top, filling all structures on the device layer. Using a reactive ion etching (RIE) tool (Oxford PlasmaPro NPG 80), the resist is partially removed by exposing only the top SiO_2_ surface, which was later removed mainly by another RIE process followed by buffered hydrofluoric acid (BHF) wet etching for the last few nanometers (to not affect the surface roughness). To pattern the metal pads on the top surface, another photolithography step with the corresponding mask was performed, followed by evaporation (Evatec BAK501 LL) of a 5 nm Chrome adhesion layer and a 60 nm gold layer. The excess metal was removed via a lift-off process by immersing the wafer in acetone. For the backside etching of the handle wafer, the last photolithography step was performed using the third mask with a tool that enabled backside alignment (Su¨ss mask aligner MA8) and using a thicker resist (Microchemicals AZ4562) to prevent undesired removal of the resist due to the long etching process. A last DRIE process was performed to etch the optical windows on the handle wafer. A depth of 482.5 µm needed to be etched until the process was selectively stopped by the BOX. Finally, the channels were sealed by anodic bonding of the perforated cover glass to the SOI wafer at 450 °C and 1100 V for ~15 min with a Su¨ss Substrate Bonder SB8e. The wafer was subsequently diced via an ADT ProVectus LA 7100 into single chips.

### Electrical characterization

The electrical resistance measurements of two adjacent electrodes under a DC load were measured with an Agilent B1500A Parameter Analyzer by applying a voltage sweep from 0 to 100 V and measuring the current drained. The measurements for electrochemical impedance spectroscopy were performed with a Potentiostat Autolab PGSTAT302N by sweeping the frequency of a 1 V AC signal from 0.01 Hz to 100 kHz and using a solution of 0.1 M KCl as the electrolyte, which was filled in the microfluidic channel.

### Microfluidic setup

The microfluidic interface was designed with CATIA V5R20 and 3D printed by stereolithography using Formlabs Form 3. The aluminum parts of the assembly were machined with a CNC tool. Spring-loaded contact probes were purchased from FeinMetall. The interface had ports compatible with M6 microfluidic fittings. The fluidic connections were further sealed using Markez FFKM O-rings and gaskets. For experimentation, the interface was mounted on an inverted microscope and connected to syringes or vials via PEEK tubing. The syringes were actuated via neMESYS (Cetoni Gmbh) syringe pumps. An arbitrary waveform generator (UTG2025A from Uni-Trend) was used to power the electrodes. Videos of the device operation were obtained using a high-speed camera (VEO E-340 L from Vision Research) connected to the microscope. For the picoinjection and deflection experiments, water-in-oil droplets was previously generated and collected using a standard flow-focusing device. Evagreen oil from Bio-Rad was used as the continuous phase, as well as additional spacing oil during operation. The devices were treated with oxygen plasma for 1 min and pretreated with Sigmacote (a siliconizing reagent for glass and other surfaces) for 5 min before utilization.

### FADS experiment

Two different droplet populations containing either green fluorescent Alexa Fluor 488 dye (Alexa, 10 µM) or red fluorescent sulforhodamine B dye (SRB, 10 µM) were produced using a standard PDMS-based flow-focusing device. A mixture of the two droplet populations containing 15% Alexa droplets and 85% SRB droplets was used. The droplet mixture was pumped into our silicon-based sorting chip with a flow rate of 15 µL/h, and the flow rate for the spacing oil was set to 100 µL/h, leading to a droplet rate of over 600 droplets per second (0.6 kHz). The fluorescence signals were recorded by DropSort software (Dropsort.eu). A sorting gate was set such that the droplet population with high green and low red fluorescence signals was sorted. Several samples from the droplet populations before and after sorting, with more than 1000 droplets each, were via an image recognition program.

### Online content

Videos from the microfluidics experiments performed can be found online, as well as the full lithographic mask design set (GDS file) and the STL files for 3D printing of the microfluidic interface. The data supporting the findings of this study are available from the corresponding authors upon request.

## Supplementary information

Fabrication Information

Fluid Mount 3D Body

FADS video 1

FADS video 2

Picoinjection 0V

Picoinjection 7V

Deflection 0V

Defelction 15 V

Deflection from OFF to ONN

## References

[1] Nguyen, N.-T., Wereley, S. T. *Fundamentals and Applications of Microfluidics* (Artech House, 2002).

[2] Squires TM, Quake SR (2005). Microfluidics: fluid physics at the nanoliter scale. Rev. Mod. Phys..

[3] Payne EM, Holland-Moritz DA, Sun S, Kennedy RT (2020). High-throughput screening by droplet microfluidics: perspective into key challenges and future prospects. Lab Chip.

[4] Mashaghi S, Abbaspourrad A, Weitz DA, van Oijen AM (2016). Droplet microfluidics: a tool for biology, chemistry and nanotechnology. Trends Anal. Chem..

[5] Rothbauer M, Zirath H, Ertl P (2018). Recent advances in microfluidic technologies for cell-to-cell interaction studies. Lab Chip.

[6] Berthier, J. & Silberzan, P. *Microfluidics for Biotechnology* (Artech House, 2006).

[7] Joensson HN, Svahn HA (2012). Droplet microfluidics - A tool for single-cell analysis. Angew. Chem. Int. Ed..

[8] Lorenz M M, Dejan B (2009). Novel trends in high-throughput screening. Curr. Opin. Pharmacol..

[9] Mattanovich D, Borth N (2006). Applications of cell sorting in biotechnology. Microb. Cell Factories.

[10] Mair B (2019). High-throughput genome-wide phenotypic screening via immunomagnetic cell sorting. Nat. Biomed. Eng..

[11] Stuart T, Satija R (2019). Integrative single-cell analysis. Nat. Rev. Genet..

[12] Huebner, A. et al. Quantitative detection of protein expression in single cells using droplet microfluidics. *Chem. Commun*. 1218–1220 (2007). 10.1039/b618570c 17356761

[13] Kaushik AM, Hsieh K, Wang T-H (2018). Droplet microfluidics for high-sensitivity and high-throughput detection and screening of disease biomarkers. Wiley Interdiscip. Rev. Nanomed. Nanobiotechnol..

[14] Zhu P, Wang L (2017). Passive and active droplet generation with microfluidics: a review. Lab Chip.

[15] Shembekar N, Hu H, Eustace D, Merten CA (2018). Single-cell droplet microfluidic screening for antibodies specifically binding to target cells. Cell Rep..

[16] Vallejo D, Nikoomanzar A, Paegel BM, Chaput JC (2019). Fluorescence-activated droplet sorting for single-cell directed evolution. ACS Synth. Biol..

[17] Love JC, Anderson JR, Whitesides GM (2001). Fabrication of three-dimensional microfluidic systems by soft lithography. MRS Bull..

[18] Oyama TG, Oyama K, Taguchi M (2020). A simple method for production of hydrophilic, rigid, and sterilized multi-layer 3D integrated polydimethylsiloxane microfluidic chips. Lab Chip.

[19] Monahan J, Gewirth AA, Nuzzo RG (2002). Indirect fluorescence detection of simple sugars via high-pH electrophoresis in poly(dimethylsiloxane) microfluidic chips. Electrophoresis.

[20] Shin YS (2003). PDMS-based micro PCR chip with parylene coating. J. Micromech. Microeng..

[21] Aymerich M, Gomez-Varela A, Alvarez E, Flores-Arias M (2016). Study of different sol- gel coatings to enhance the lifetime of PDMS devices: evaluation of their biocompatibility. Materials.

[22] Tucher N, Höhn O, Hauser H, Müller C, Bläsi B (2017). Characterizing the degradation of PDMS stamps in nanoimprint lithography. Microelectron. Eng..

[23] Piruska A (2005). The autofluorescence of plastic materials and chips measured under laser irradiation. Lab Chip.

[24] Ahn K (2006). Dielectrophoretic manipulation of drops for high-speed microfluidic sorting devices. Appl. Phys. Lett..

[25] Rao L (2015). One-step fabrication of 3D silver paste electrodes into microfluidic devices for enhanced droplet-based cell sorting. AIP Adv..

[26] Sciambi A, Abate AR (2014). Generating electric fields in PDMS microfluidic devices with salt water electrodes. Lab Chip.

[27] Sesen M, Alan T, Neild A (2017). Droplet control technologies for microfluidic high throughput screening (*µ*HTS). Lab Chip.

[28] Unger MA, Chou H-P, Thorsen T, Scherer A, Quake SR (2000). Monolithic microfabricated valves and pumps by multilayer soft lithography. Science.

[29] Ding X (2013). Surface acoustic wave microfluidics. Lab Chip.

[30] Cui W, Yesiloz G, Ren CL (2020). Microwave heating induced on-demand droplet generation in microfluidic systems. Anal. Chem..

[31] Kemna EWM, Segerink LI, Wolbers F, Vermes I, van den Berg A (2013). Label-free, high-throughput, electrical detection of cells in droplets. Analyst.

[32] Ahn K, Agresti J, Chong H, Marquez M, Weitz DA (2006). Electrocoalescence of drops synchronized by size-dependent flow in microfluidic channels. Appl. Phys. Lett..

[33] Abate AR, Hung T, Mary P, Agresti JJ, Weitz DA (2010). High-throughput injection with microfluidics using picoinjectors. Proc. Natl Acad. Sci. USA.

[34] Brouzes E (2009). Droplet microfluidic tech- nology for single-cell high-throughput screening. Proc. Natl Acad. Sci. USA.

[35] Mazutis L (2009). Multi-step microfluidic droplet processing: kinetic analysis of an in vitro translated enzyme*.*. Lab Chip.

[36] Park J (2005). An efficient cell separation system using 3D-asymmetric microelectrodes. Lab Chip.

[37] Lewpiriyawong N, Yang C (2012). AC-dielectrophoretic characterization and separation of submicron and micron particles using sidewall AgPDMS electrodes. Biomicrofluidics.

[38] Niu X, Zhang M, Peng S, Wen W, Sheng P (2007). Real-time detection, control, and sorting of microfluidic droplets. Biomicrofluidics.

[39] Nuttawut L, Chun Y, Cheong LY (2010). Continuous sorting and separation of microparticles by size using AC dielectrophoresis in a PDMS microfluidic device with 3-D con- ducting PDMS composite electrodes. Electrophoresis.

[40] Saucedo-Espinosa MA, Dittrich PS (2020). In-droplet electrophoretic separation and enrichment of biomolecules. Anal. Chem..

[41] Iliescu C, Yu L, Tay FE, Chen B (2008). Bidirectional field-flow particle separation method in a dielectrophoretic chip with 3D electrodes. Sens. Actuators B Chem..

[42] Choi J-W (2010). 3-dimensional electrode patterning within a microfluidic channel using metal ion implantation. Lab Chip.

[43] McIntyre D, Lashkaripour A, Densmore D (2020). Rapid and inexpensive microfluidic electrode integration with conductive ink. Lab Chip.

[44] Woolley AT (1996). Functional integration of PCR amplification and capillary electrophoresis in a micro-fabricated DNA analysis device. Anal. Chem..

[45] Puebla Hellmann, G. F., Monserrat Lopez, D., Drechsler, U., Mayor, M. & Lörtscher, E. Microfluidic devices with electrodes formed as physically separated sections of microchannel side walls. US patent 2022/135399 A1 (2022).

[46] Celler GK, Cristoloveanu S (2003). Frontiers of silicon-on-insulator. J. Appl. Phys..

[47] Lang W (1996). Silicon microstructuring technology. Mater. Sci. Eng.,.

[48] Deal BE, Grove AS (1965). General relationship for the thermal oxidation of silicon. J. Appl. Phys..

[49] Xiao, H. *Introduction to Semiconductor Manufacturing Technology* (SPIE Press, 2012).

[50] Bandarenka AS (2013). Exploring the interfaces between metal electrodes and aqueous elec- trolytes with electrochemical impedance spectroscopy. Analyst.

[51] Temiz Y, Lovchik RD, Kaigala GV, Delamarche E (2015). Lab-on-a-chip devices: how to close and plug the lab?. Microelectron. Eng..

[52] Priest C, Herminghaus S, Seemann R (2006). Controlled electrocoalescence in microfluidics: targeting a single lamella. Appl. Phys. Lett..

[53] Herminghaus S (1999). Dynamical instability of thin liquid films between conducting media. Phys. Rev. Lett..

[54] Eow JS, Ghadiri M (2003). Drop-drop coalescence in an electric field: the effects of applied electric field and electrode geometry. Colloids Surf. A Physicochem. Eng. Asp..

[55] Yuan H (2018). Picoinjection-enabled multitarget loop-mediated isothermal amplification for detection of foodborne pathogens. Anal. Chem..

[56] Sjostrom SL, Joensson HN, Svahn HA (2013). Multiplex analysis of enzyme kinetics and inhibition by droplet microfluidics using picoinjectors. Lab Chip.

[57] Ahmed H, Stokke BT (2021). Fabrication of monodisperse alginate microgel beads by microfluidic picoinjection: a chelate free approach. Lab Chip.

[58] Pohl, H. A. in *Dielectrophoresis: The Behavior of Neutral Matter in Nonuniform Electric Fields* (ed. Mauro, A.) (Cambridge Univ. Press, 1978).

[59] Kintses B (2012). Picoliter cell lysate assays in microfluidic droplet compartments for directed enzyme evolution. Chem. Biol..

[60] Neun S (2022). Functional metagenomic screening identifies an unexpected B-glucuronidase. Nat. Chem. Biol..

[61] Debon A (2019). Ultrahigh-throughput screening enables efficient single-round oxidase remodelling. Nat. Catal..

[62] Colin P-Y (2015). Ultrahigh-throughput discovery of promiscuous enzymes by picodroplet functional metagenomics. Nat. Commun..

