# Supplementary material for: Direct electrification of silicon microfluidics for electric field applications
Source: Microsyst Nanoeng. 2023 Jun 19;9:81. doi: 10.1038/s41378-023-00552-w (PMC10277806; doi:10.1038/s41378-023-00552-w)
Supplement: Supplementary file 1 — Fabrication Information [file 41378_2023_552_MOESM1_ESM.pdf]

Photolithography Masks:

All designs are contained in the file *SupInfo_Masks_Designs.gds*

Layer	Design Description	Subsequent Process
0/1	Microfluidic channels and electrodes pattern	DRIE as deep as SOI wafer device layer
1/1	Backside openings for view-ports openings	DRIE as deep as SOI handle layer
4/1	Metallic pads to contact electrodes	Metal deposition on top of device layer
110/1	Wafer and mask outline	Not to be used

Microfluidic interface:

The file *Interface_body.stl* contains the design to be 3D printed, while all the pieces to be machined by CNC are included in the zip folder Aluminum_parts.zip

File	Description	Recommended Fabrication
Interface_body.stl	Main body of the microfluidic interface	3D printing with Formlabs Form 2 SLA Printer. Using white resin (FLGPWH04) and layer thickness of 0.1 mm
Cover_Big.stl	Big piece to be screwed on one side of the top part of the microfluidic interface, allowing the cover to open and close	Aluminum milling by CNC
Cover_Small.stl	Small piece to be screwed to the opening at the center of the Big Cover	Aluminum milling by CNC
Cover_Bottom.stl	Flat cover to be screwed to the bottom of the microfluidic interface to protect the electrical connections	Aluminum milling by CNC
Cover_Side_A.stl	Flat pieces to be screwed on the front side of the microfluidic interface to hold the SMB coaxial connectors	Aluminum milling by CNC
Cover_Side_B.stl	Flat piece to be screwed on the rear side of the microfluidic interface to hold the SMB coaxial connectors	Aluminum milling by CNC

**Please refer to the main paper for further details and specifications.*
